# Can Contextualization Increase Understanding During Man-Machine Communication? A Theory-Driven Study

**DOI:** 10.2174/1874431100802010082

**Published:** 2008-05-22

**Authors:** L.L Alpay, J Verhoef, D Te’eni, H Putter, P.J Toussaint, J.H.M Zwetsloot-Schonk

**Affiliations:** 1 Clinical Informatics Group, Department of Medical Statistics and Bioinformatics, Leiden University Medical Center (LUMC), Leiden, The Netherlands; 2 Department of Physical Therapy, Leiden University Medical Center (LUMC), Leiden, The Netherlands; 3 Faculty of Management Tel Aviv University, Tel Aviv, Israel; 4 Medical Statistics Group, Department of Medical Statistics and Bioinformatics, Leiden University Medical Center, Leiden, The Netherlands; 5 Department of Computer and Information Science, Norwegian University of Science and Technology (NTNU), Trondheim, Norway

**Keywords:** Internet, communication, consumer health information, contextualization of information, information retrieval.

## Abstract

The Internet offers unlimited possibilities for finding health information. However, the user is often faced with the problem of understanding it. Contextualization has a role to play in enhancing the user’s comprehension. We report on a study which addresses this issue, using a theoretical model of communication whose central theme is that of context. A randomized controlled experimental design was chosen, using as a test-bed the website SeniorGezond we had previously developed. The study was composed of a pre-test, the intervention with the website and a post-test. Participants (n=40) were randomly assigned to exposure or no exposure to contextualization with the website. Results show that contextualization increases understanding for non-knowledgeable users. Furthermore, the participant’s cognitive style was found to be a significant factor on understanding. We also found that participants bring their own contexts such as social context and psychological context to support their understanding.

## INTRODUCTION

### Information Search on the Internet & Challenges for the Consumer of Health Information

The Internet has become a popular tool which offers unlimited possibilities for finding health information. Indeed, a new health consumer identity is emerging, sometimes referred to as the "on-line self-helpers” [1]. In their quest for health information, users are usually faced with several challenges, namely that of searching for relevant information, of accessing a good quality of information, and of understanding the information retrieved. Health professionals are also experiencing an identity shift, moving from the authoritative figure to that of facilitator [2]. Professionals’ information and knowledge are, among other ways of distribution, commonly mediated on the Internet *via *health related websites. The health information provider has often limited insight about the user's understanding of the information given by means of these health-related websites.

Information understanding can put strong cognitive demands on the users, especially the older users, and is closely related to that of the construct of contextualization of information. The notion of context appears in several disciplines including cognitive psychology and computer science. Context in our research is studied within the framework of communication, specifically man-machine health communication. Durnell-Cramton [3] has shown that the lack of contextualization is a common problem in communication under dispersed and technology mediated conditions. Contextualization is about providing supportive information to explain a core message (in this case a health message). The contextualization of the information can help improving the user's understanding and sustain an effective man-machine communication [4, 5]. In particular, contextualization of information can help to reduce the user’s cognitive distance (i.e. the difference in knowledge between the user and the website as the provider of information). In this paper, we report on a study investigating the impact of context on the user's understanding.

### Te’eni Model of Communication

In order to investigate the impact of contextualized information on the user’s understanding of the retrieved information, we were guided by the model of communication proposed by Te’eni [4] whose central idea is that of communication within a well-defined context. In his model of communication, Te'eni introduces the notion of *communication complexity* as the communicator's sense of the cognitive effort needed to ensure effective communication. Effective communication results from the use of cognitive resources to overcome the difficulties in understanding and uncertainty about the message. Another construct of this model is that of *mutual understanding (MU) *which requires that the communication be comprehensible according to the sender's meaning and the user's cognitive capacity.

**Fig. (1):**
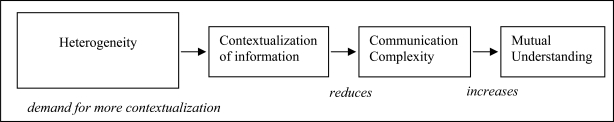
Te’eni model of communication.

Central to the model is the idea of *context*. Contextualization requires cognitive resources and is seen as a communication activity aimed at increasing mutual understanding by reducing complexity. In other words, contextualization is about providing contextual information to reduce its complexity and thereby support understanding of a core message. Te'eni found that communication complexity is due to the intensity of information, the multiplicity of views, the incompatibility between representations and use of information, and affected by the heterogeneity of the communicators involved, and so by the terminology they used to communicate. Heterogeneity triggers communicators to incorporate more contextual information in order to improve mutual understanding. Contextualization, if effective, reduces communication complexity and thereby increases mutual understanding (see Fig. **1**).

The Te’eni model comprises six hypotheses around the various aspects of communication complexity, heterogeneity, contextualization and mutual understanding [4]. Given the scope of this study, we selected the hypothesis no. 5 which states that**:** *The impact of contextualization on mutual understanding is contingent on heterogeneity: for heterogeneity, contextualization leads to higher mutual understanding, but for homogeneity, it does not.* This hypothesis was adapted for our study reported here and formulated as followed: *The impact of contextualization on mutual understanding depends on the cognitive distance between the user’s knowledge of the relevant health topic and the knowledge available in the website. *The hypothesis states that a user with a low cognitive distance (i.e. a person knowledgeable in the field) will have a high level of mutual understanding whether or not they use contextualization. Conversely, a user with a high cognitive distance (e.g. a non-knowledgeable person) is expected to have a low mutual understanding with no contextualization (e.g. a missing level of information, a missing link between the levels of information) and a higher mutual understanding with contextualization (see Table **1**). The model of communication proposed by Te’eni has been tested in certain domains such as engineering (machine assembly) but has not yet been used in the healthcare area.

### Objective of the Study

The aim of the study was twofold: 1) To test the hypothesis on the impact of contextualization when searching for health information in an informative website, and 2) to uncover types of contextualization which are relevant to the users searching for health related information.

## METHODS

### The Test-Bed: SeniorGezond

The website SeniorGezond (http://www.seniorgezond.nl) was used as the test-bed for our investigation. This website has been developed in our institution and is dedicated to the older people and their care givers in the area of fall prevention [6,7]. Falling is a major health problem within the older population.

About one third of the older people fall at least once a year, and due to demographic ageing, the group of older people will expand [8].

The website SeniorGezond is built to inform and educate the older people and their caregivers about the risks of falling and present them with possible actions that they can take to prevent falls. The website has no commercial purpose and is purely informative. While the website provides information about products and services, it is up to the website visitor to make an informed decision/choice about what products and services suit him or her best. Whether the user takes action and buys such a product is beyond this study.

### Manipulation of the Contextualization

**Fig. (2):**
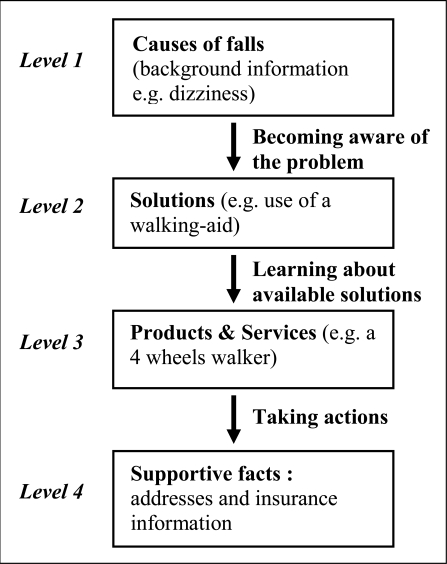
Information structure in the SeniorGezond website.

The information within the website is structured around problems of fall incidences, and contains four levels of information (see Fig. **2**). Navigation within the information structured is done using pull-down menus. The top level Causes of falls includes frequently occurring problems in the domain of fall incidences e.g. dizziness. The second level Solutions focuses on possible interventions and advices associated with the causes of falling e.g. use of a walking aid. Solutions are supported by the third level Products & Services e.g. walking aids, fitness programmes. There is no overlap in information between level 2 and level 3. Level 3 shows pictures or descriptions of the products and services; the rationale for choosing a product or a service is given in level 2. From the Products & Services level, users can access the Supportive Facts which makes up the fourth and lowest level of the information trees. Supportive Facts contain addresses about where to purchase products and services, as well as insurance related information. The four levels take the user from general to specific information.

Manipulation of the contextualization was achieved by removing the Solutions level from the navigation (2^nd^ level) as well as cross-links to the other levels. This level embeds relevant contextualization since its acts as a buffer between the background information level and the Products and Services level. In particular, the Products and Services level only gives *description*s about products such as walking-aids and services such as sport and physical exercises but no information about the reasons one should select such a product or service (given in level 2 Solutions).

### Participants

Forty participants were recruited via advertisements in local newspapers and via personal contacts. Prerequisites for the selection included familiarity with Internet usage and not being a healthcare professional.

### Design

**Fig. (3):**
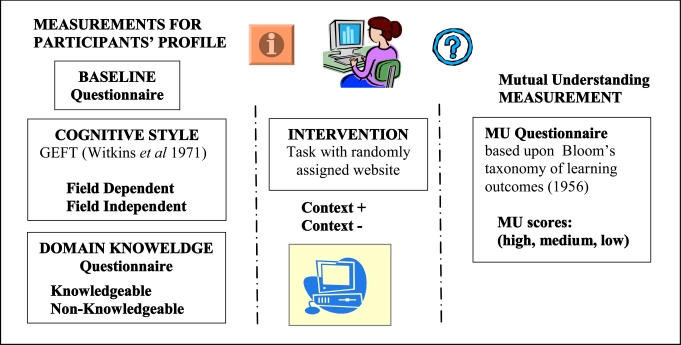
Study design.

A randomized controlled experimental design was chosen. The study was composed of a pre-test for gathering participants' profile, the intervention with the website and a post-test for measuring participants' mutual understanding (see Fig. **3**). The exposure to contextualization was randomly distributed amongst the participants. In this design, context within the website was the independent variable while participant’s MU was the dependent variable. Possible confounding variables which were considered include participants’ domain knowledge and participants’ cognitive style.

In the gathering phase, participants’ characteristics (baseline instrument) and their knowledge about the domain (domain knowledge instrument) were collected as well as their cognitive styles *via *the GEFT test [9].

During the intervention phase, participants with the context and no-context manipulated website were given the same task to carry out. Participants were given the task of finding an appropriate walking aid for a fictive older family member who has a problem of dizziness

To complete the task, all participants (independently whether they themselves had previously experienced a fall) were asked 1) to select an appropriate walking-aid for this family member and 2) to explain the reasons why (using structured questions, for example regarding the distance one can walk with the selected walking-aid). Participants were instructed to read the information from the website (rather than just guessing). In the last phase, participants were tested for their understanding of health information they had found in the website.

### Use and Development of the Instruments

Four instruments were needed for data collection and hypothesis testing: Three instruments were self-developed (Baseline instrument, Domain Knowledge instrument and Mutual Understanding instrument); a cognitive style instrument (Group Embedded Figure Test; GEFT) from the field of psychology was used unchanged [9].

#### Baseline Instrument

Participants’ characteristics were collected *via *a self-developed questionnaire. The questionnaire was built by the authors based on previous work, complemented with information found in the literature. As a basis we took the questionnaire we made for our usability testing of the final version of the website. From this questionnaire, we extracted four questions about participants’ demographics as well as their Internet usage and previous falls. We then added four questions to obtain more detailed information about their Internet usage and previous falls.

#### Domain Knowledge Instrument

Participants’ knowledge about the domain was also collected *via *questionnaires. There is no standard questionnaire to measure knowledge in the domain of fall incidences. Such a questionnaire was developed in our group as part of a qualitative evaluation of the website SeniorGezond [7]. The original questionnaire includes ten questions with regards to the attitude towards fall prevention, general facts about falls, causes of falls, and consequences of falling. We re-used the questionnaire and adapted it for the study reported here. In particular we added six questions regarding medicine intake, urine incontinency, dizziness & balance problem, and about walking aids. The content of the questionnaire was agreed upon by two health professionals from the Leiden University Medical Centre (LUMC), who are considered to be experts in the field of fall prevention.

#### Mutual Understanding Instrument

Understanding can be defined as "the ability to think and act flexibly with what one knows" [10]. In our study, MU refers to the user's understanding (comprehension) of the information he/she has found. In our experiment, MU is not about a person-to-person understanding (one user understanding another user) but rather a person-to-system understanding.

The outcome parameter of mutual understanding (MU) was developed specifically for the study and is based on Bloom’s taxonomy of learning outcomes [11]. While using the website to perform the task, participants are in a learning situation in that they have to acquire knowledge about fall prevention. Thus, we chose to measure MU from a learning perspective and draw from the literature on lifelong learning, distance education and teaching. To our knowledge, this is an innovative and unexplored way to apply this taxonomy which includes six hierarchical levels for learning outcomes: knowledge, comprehension, application, analysis, synthesis, and evaluation. The taxonomy provides a useful structure in which to categorize and prepare test questions:

- *Level 1 knowledge:* recalling factual information.
- *Level 2 comprehension:* explaining the meaning of information, association of concepts, differentiation.
- *Level 3 application:*  using information in concrete situations. Application involves the recall of knowledge in combination with comprehension to describe a new situation.
- *Level 4 analysis:*  breaking down a whole into its constituting components. Analysis involves analyzing data at hand. It involves for example recognizing unstated assumptions and error in reasoning, making inferences, evaluating relevance of the data.
- *Level 5 synthesis:* putting parts together to form a new and integrated whole. Synthesis involves higher skills of information formation and processing. Synthesis typically involves creating a new product or combining of ideas to form a new whole.
- *Level 6 evaluation:* making judgments. Typically evaluation is concerned, for example, with making value decision about issues, resolving controversies or differences of opinion, and developing opinions and judgments.

The MU instrument we developed contained the first four levels of the taxonomy with a total of seven problems: two at the level 1 knowledge; two at the level 2 comprehension; two at level 3 application and one at level 4 analysis. The MU instrument took the form of open questions, statements measured on a 3-point Likert Scale for agreement/disagreement, and open questions based on a case description. We did not include the last two levels since they are more relevant for testing professionals (or to be professionals) in the field rather than lay people (our targeted group). The mutual understanding instrument was reviewed by two professionals from the rheumatology department at LUMC. They insured the accuracy of the casus presented to the participants and the appropriate level of difficulty of the task. The total score of the questions for all the categories makes up the MU score. Three levels of score were pre-defined, namely Low (0-15), Medium (16-31) and High (32-42).

#### Cognitive Style Instrument

Participants’ cognitive style was identified using the psychological test, the Group Embedded Figure Test (GEFT) instrument [9]. This test determines whether a person is field dependent (i.e. approaches a problem in a holistic and global way) or field independent (i.e. approaches a problem in an analytical, deterministic way). Cognitive styles refer to the preferred way an individual organizes, filters, transforms and processes information. A person’s cognitive style is determined by the way the person takes in the environment in which he is embedded. Findings from research suggest that users with different cognitive styles develop different strategies and tactics when seeking information on the Web e.g. [12].

Although information seeking depends on the operation of the same basic cognitive processes, not every person exhibits similar information seeking tactics. Information seeking behavior is highly variable because it is associated with elements or characteristics that are significantly different from one individual to the other.

The validity of the GEFT instrument was established and reported by [9] based on its parent test, the Embedded Figures Test. Moreover, the GEFT is a standardized instrument with a reliability estimate of .82 (reported by Witkin *et al* [9]). The test booklets and the scoring keys were acquired *via *Mind Garden Inc. (http://www.mindgarden.com). The translation into Dutch was done, following the translation methodology advocated by Eremenco [13] and in agreement with Mind Garden Inc.

### Methods for Data Analysis

#### Scoring of the Questions

Questions within the various questionnaires combined open questions with multiple choice questions. These latter were easily scored (correct or incorrect). For each open question, a set of expected answers was created and the participant’s answer was matched against these expected answers. Open questions were scored as correct, incorrect (no answer, wrong answer) or correct but not complete. Scoring was first done by the first author and then validated by the second author.

#### Quantitative Analysis

Data collected was analyzed quantitatively using the SPSS 14.0 statistical package. Statistical analysis included basic frequencies, Chi square test or Fisher’s exact test, and Student’s t-test where appropriate. A two-way between-groups analysis of variance (Two-way ANOVA) was conducted to explore the impact of type of website; the level of knowledge, and the type of cognitive style on the levels of mutual understanding.

#### Qualitative Analysis

In complement, qualitative analysis of the task performed and of the contents of MU was done using NVivo software^1^. Participants’ answers to complete the task, and to the problems in the MU questionnaires were transcribed and coded. Coding was first done by the first author and then checked with the second author for agreement and resolution of any discrepancy. A set of basic categories was first established based on the contents of the questions. Subsequent coding categories were data-driven.

## RESULTS FROM QUANTITATIVE ANALYSIS

### Participants

Forty participants took part, half of whom were randomly given exposure to contextualization and the other half had no contextualization exposure. The total group of participants (n = 40), before the random assignment, had a mean age of 56,7 (SD 14,4) years old (varied from 20 to 80 years old), and there were more female (n=24) than male (n=16) participants. The educational level^2^ of the participants was 12,5 % (n=5) low education level, 37,5 % (n=15) medium education level and 50% (n=20) a high education level. Most of the participants used Internet on a daily basis (67.5%; n=27) or at least once per week (31.5%; n=13). Almost half of the number of participants (47,5%; n=19) looked for health-related information on the Internet for at least once per month. In 57,5% (n=23) of the participants someone in his environment (such as family members, relatives or friends) has had a fall accident and 25% (n=10) of the participants has had a fall incident themselves.

The frequency of Internet usage with respect to participants’ age group is shown in Fig. (**4**).

After the randomization there were no statistical significant differences between the two groups regarding age, sex, educational level, Internet usage, frequency of searching health related information, and fall incidences (Mann Whitney U test, Chi square test or Fisher’s exact test, where appropriate). Data were collected between November 2006 and March 2007.

### Knowledgeable About the Domain

Fig (**5**) shows that the mean domain knowledge of all the participants (n=40) was 57% (SD 14%). Experts (n=12) from LUMC also completed the domain knowledge questionnaire and as expected scored higher than the participants (mean domain knowledge was 75% (SD 11%). We defined participants to be knowledgeable in the field of falling (threshold) when they have a score higher than 57% (21 participants >57%; 19 participants < 57%).

### Mutual Understanding

**Fig. (6):**
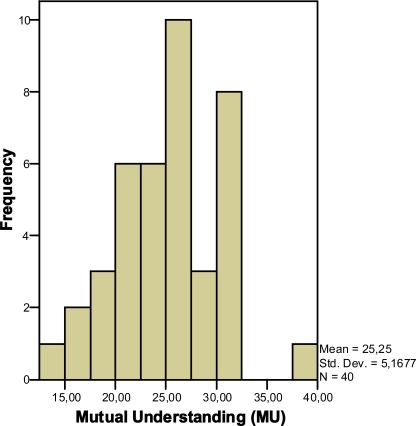
Distribution of MU scores.

**Fig. (7):**
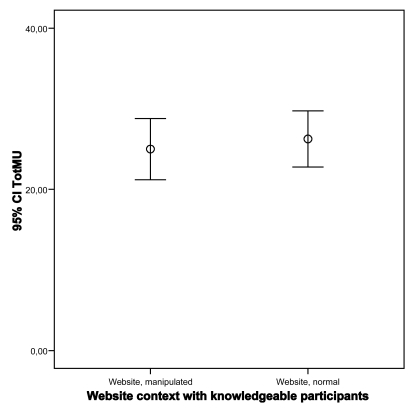
Impact of website on MU for knowledgeable participants.

**Fig. (8):**
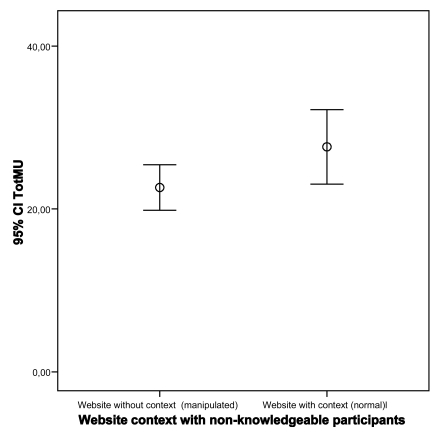
Impact of website on MU for non-knowledgeable participants.

Analysis of participants’ MU shows that participants mainly scored in the medium range (see Fig. **6**). Contrary to our expectations, almost no low or high MU score was found. Results show that the impact of the manipulated website on MU only for non-knowledgeable participants is statistically significant (p=0.60 and p=0.04; knowledgeable and non-knowledgeable, respectively; Student’s t-test). In other words contextualization tends to increase understanding for non-knowledgeable users (see Figs. **7**,**8**).

Two-way ANOVA revealed neither statistically significant main effects for type of website (F=3.76; df=1; p=0.06) and level of knowledge (F=0.09; df=1; p=0.76) nor their interaction effects (F=1.35; df=1; p=0.25).

### Cognitive Styles

**Fig. (9):**
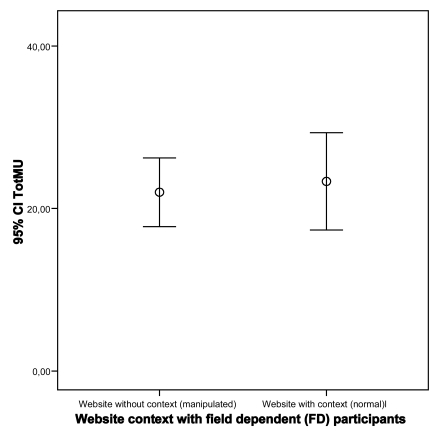
Impact of website on MU for Field Dependent participants.

**Fig. (10):**
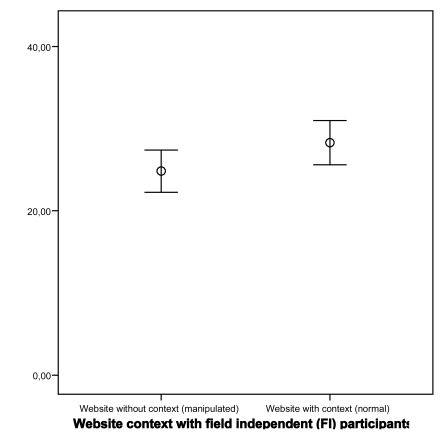
Impact of website on MU for Field Independent participants.

Results from the GEFT indicated that a majority of the participants (65%, n=26) can be classified as Field Independent (FI) and the rest (35%, n=14) as Field Dependent (FD). Further results showed that the impact of the type of cognitive style (FD and FI) on MU is not statistically significant for both types of websites (p=0.65 and p=0.06; FD and FI respectively; Student’s t-test). In other words participants’ different cognitive styles do not have an impact on mutual understanding in case of using a normal website as well as using a manipulated website (see Figs. **9**,**10**).

Two-way ANOVA, however, revealed a statistically significant main effect for the type of cognitive style on mutual understanding (f=6.08; df=1; p=0.02) with a large effect size (Partial Eta Squared=0.15). The main effect for the type of website (F=2.30; df=1; p=0.14) and the interaction effect (F=0.45; df=1; p=0.51) did not reach statistical significance.

## RESULTS FROM QUALITATIVE ANALYSIS

### Contextualization Themes

Transcripts from the MU instrument helped to identify participants’ contextualization themes. Results indicate that participants bring their own contexts when searching and deciding which information is relevant. We have identified two types of contexts, namely social context and psychological context.

#### Social Context

In our study where the task focused on the choice of a walking aid, social factors seem to play a contextual role when deciding which one can be used. The stigma of being seen as old and handicap or invalid seems to influence the choice of a stick instead of a wheeled walker whereas the latter may actually be safer for the person’s physical abilities.

The participants’ quotes (translated from Dutch) illustrate this perception (see Table **2**, Examples 1 to 3). Conversely, a person with a wheeled walker may be given way to cross the road first, providing a sense of social acceptance and possibly respect and politeness (see Table **2**, Example 4).

Furthermore, outside hazards within the social environment is also a relevant context influencing the choice of a walking aid. Typically someone with a wheeled walker will often put her/his bag on the basket provided in front of the walking aid. In this situation, the older person can be an easy target of a thief (see Table **2**, Example 5). While a 4 wheeled walker may be seen as putting the older person in a vulnerable situation, other types of walking aid are on the contrary seen as an added value in case of danger (see Example 6 in Table **2**).

#### Psychological Context

A number of psychological factors were found in relation to the use of a walking aid. There seems to be a strong need to be and remain in control of what one can do despite some physical limitations (see examples 7, 8 and 9 in Table **3**). Psychological factors also reflect current concerns of security and fear of falling (see examples 10 & 11 in Table **3**).

## DISCUSSION & FURTHER WORK

### Theoretical Framework

Results from this empirical investigation corroborate the hypothesis from the Te’eni model, namely, that exposure to contextualization increases understanding for non-knowledgeable users. However, care should be taken when interpreting this result. Indeed there maybe two possible factors which have affected the outcomes:

1. *Participants’ MU threshold:* almost no low or high MU score was found. The participants’ outcome measure of MU fell mainly within the median score threshold.
2. *Distribution of FI vs FD participants:* More participants were identified as Field Independent (n=26) than Field Dependent (n=14). This may partly account for the participants’ outcomes of the MU scores.

As stated in the introduction, the communication model of Te’eni is complex and comprises a set of six hypotheses. Our study has been based on one of those hypotheses. Work on contextualization will need in the future to take into account additional interactions of the different components (see Fig. **1**) as foreseen within the remaining hypotheses. In particular, of immediate interest are hypothesis #1 “Greater heterogeneity of communicators leads to more contextualization”, and hypothesis #3 “More contextualization reduces communication complexity”.

It should also be stressed that the website was not developed with functionalities of contextualization in mind. As stated in the Methods section, the website was used as a test-bed for our current investigation. The contents was structured using the Precaution Adoption Process Model, a health behavioral change stage theory [14]. Users need information that fit the stage they are in, and that helps them to move to the next stage of the model where eventually, they might take and maintain action to prevent from falling [15]. The stages of the model were translated into a users’ information need and thereupon converted into the information levels of SeniorGezond.

### Cognitive Styles

Kim *et al.* [12] have shown that the cognitive style (Field Independent / Field Dependent) plays a role when searching for information on the Internet. In particular, they have found that a Field Independent person tends to perform efficient searches, and use active and analytic searches more often. Conversely, a Field Dependent person will choose tools that are salient but not necessarily useful for completing the information searching task, use Home keys more often and tend to be distracted easily. Our finding confirms that the cognitive style Field Independent / Field Dependent is a significant factor in information seeking. However, in the scope of our study, we have not examined which web functions participants used (such as keyword search or “back” button). It is certainly an aspect which needs to be investigated further. An analysis of the web logs collected from the participants who worked with the non-context version of the website would be a starting point.

Other dimensions of cognitive styles have also been examined in the field of web information seeking, but not extensively. For example, work has been carried out [16] to measure the cognitive style “verbaliser” versus “imager” in relation to information seeking on the Internet. It is clear that other dimensions of cognitive styles should not be over looked in our future investigations.

### Contexts

Results show that participants bring other non-informational contexts (namely social and psychological) when searching for answers and justify their choice. Indeed, information seeking does not occur in a vacuum but rather arises from and is conditioned by the circumstances in which the information seeker is [17]. People tend to make sense of the information they have at their disposal in the light of their own needs and their own interpretations. Wilson’s model of information behavior shows how psychological and interpersonal environmental characteristics influence a person’s information seeking process and interpretation [18]. It is therefore possible that participants in our study have relied on psychological and social factors to 'reinforce' the information they found and selected.

Furthermore, within this sphere of social and psychological contexts, social perception [19] and social judgment [20] seem to play here an important role. Social perception is the study of how we form impressions of and make inferences about other people. The central idea of social judgment theory is that attitude change is mediated by judgmental processes and effects used to persuade people. In our study, fear of being stigmatized as invalid and handicap as reported by some of our participants is a good illustration of such social perception and social judgment. Not surprisingly, these remarks of stigmatization came from the older participants. Stigma and discrimination of being old remain for the older participants a real concern. In defense against the stigma, older individuals tend to deny their old age through for example physical disguises, the attainment of active mastery in areas traditionally closed to elders, avoidance of social interactions with other age groups, or self-other identification [21]. In our study, the choice of a walking aid such as a stick which is less visible (than a 4 wheel-walker) may be seen as an example of such attitude against stigmatization.

While contextualization of information such as the interventions level of SeniorGezond enhances some participants’ understanding of the domain area, our results indicate that understanding is coupled with other cognitive abilities such as:

#### Remembering experiences with walking aids:

Some of the participants bring their own experience with walking aids, having themselves used a walking aid or knowing someone who does. Previous research has shown that users tend to choose information on the basis of familiarity rather than usefulness [22]. It is thus probable that a participant’s familiarity with 4 wheels-walkers would favor the choice of this walking aid rather than another one. In our study, participants who said to have some knowledge about walking-aids were not excluded since this did not guarantee that they had an understanding of the use of such walking-aids.

#### Transferring information to daily life situations:

Participants tend to rely on examples from daily life situations to support and confirm the information they selected. This includes for example 1) choosing a stick which is easier to use when traveling with the public transport (a bus or a train); 2) wearing good walking shoes when using a stick (participant #18); 3) watching out for dogs’ excrements so as not to fall (participant #18); 4) rejecting a wheeled walker with an open basket to avoid being robbed (participant #19).

#### Using common sense:

Some participants at times rely on common sense to provide answers from the questions posed during the task with the website and during the post-test. Common sense ideas tend to relate to events within human experience and thus commensurate with human scale. An example of such common sense reasoning is that a 4 wheel-walker “provides support” and “a means to rest on it” (participant #7, question MU no.7 on the reasons for choosing a 4 wheel-walker with regards to body’s compensations). One issue here is to what extend users' shallow mental model of the problem area (i.e. walking aids and falls) account for this. Indeed, it seems that a superficial understanding of the domain selected for the study was enough to “get by” in answering the post-test questions. These findings are closely related to how people process newly found web information. Research in social cognition [23] provides relevant insights to this problem. It shows that people tend to integrate new information into pre-established schemas, especially when that information is contrary with those pre-established schemas. These pre-established schemas tend to guide attention to new information. Moreover, people selectively attend to information that is consistent with the schema and ignore information that is inconsistent. Findings from social cognition research can certainly be a source of enrichment for our work on contextualization of information.

In recent years, there has been a shift in web information seeking research from a solely focus on cognition towards a social-cultural perspective [24]. More and more information seeking is not seen as an activity which is isolated from contextual, social and cultural factors. In other words, the individual is driven to seek information not only because of cognitive needs but also because of the necessity to satisfy affective needs created by living and working in social settings [18]. It is important that user’s behavior of web information seeking be studied from all its multiple facets such as experience, information need, affective and cognitive characteristics, and socially and culturally determined traits [25]. Our findings on contextualization of information are relevant to this problem, and can help to capture some aspects of this multiple-faceted approach.

### Contextualization Guidelines

Designing websites with functionalities of contextualization can strengthen the already existing informative functions and is a step towards getting closer to the user's own situation and preferences. However, while this top-down approach (from experts to users) is important, one should not ignore the opposite way – from users to experts – which takes into account the users' own non-informational contexts. There is a gap between the informational contexts proposed to the website visitors (such as the level of possible solutions) and the user’s non-informational contexts. This gap needs to be bridged to insure that both top-down and bottom-up approaches to contexts can be coupled in order to develop coherent contextualization guidelines. Within the setting of our study, we had chosen the whole level of interventions as context. However, it is not the unique possibility. More work needs to be carried out in order to investigate which website contexts as well as users’ contexts should be implemented in a given application. Such investigation cannot be restricted to only one website. We plan to use other existing Dutch health portals for this future investigation.

## CONCLUSIONS

Health communication mediated *via *the Internet presents many challenges to the research community. One aspect of communication which is of interest to us is that of understanding the health message being retrieved by the users. Web users increasingly tend to seek on the Internet tailored and more customized information which can fit their own personal circumstances. Our theoretically-driven investigation has been focused on the contextualization of information which plays a role in enhancing the non-knowledgeable website visitor’s understanding of the health message being delivered. This study has provided some empirical evidence in this direction. Furthermore, it is a step towards developing contextualization guidelines which can be part of design requirements for tailor-made health related websites.

## Figures and Tables

**Fig. (4):**
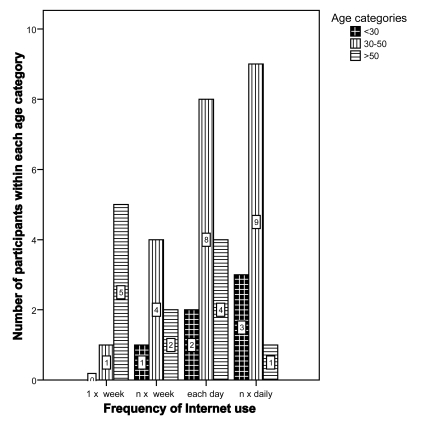
Frequency of Internet usage.

**Fig. (5):**
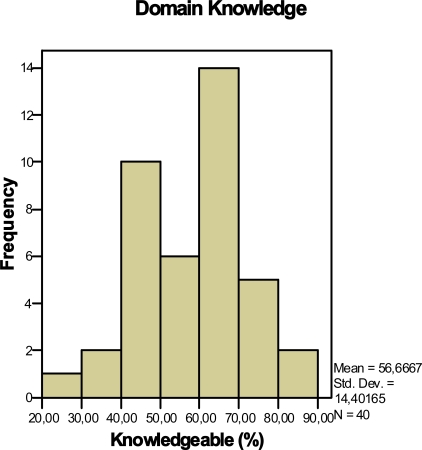
Distribution of the participants’ domain knowledge.

**Table 1.** Hypothesis Testing

Domain Knowledge	Context	Mutual Understanding
+	+	= or ↑
+	-	=
-	-	= or ↓
-	+	↑

**Table 2.** Examples of Social Contexts

** Examples about stigmatization** Example 1: “A walking stick gives sufficient support for different surfaces. The woman can carry a bag. She does not look immediately old or invalid”. (participant #5) Example 2: “The walking stick represents a ‘socially’ low barrier”. (participants #3) Example 3: “Having a stick looks friendly. Advising crutches or a wheeled walker as first choice stigmatizes”. (participant #18)
** Example about social acceptance** Example 4: “She is more visible and if needed is helped by people on the street. When crossing the road, she often gets to go first”. (participant #19)
** Example about outside hazards** Example 5: “Pity that a typical wheeled walker offers no possibility to hide what’s on it. With the existing walkers, a bag or purse can be easily stolen. It is there on display for the thief. Usually the walker falls down while the thief is already gone”. (participant #19) Example 6: “Takes little place (a stick) and can be used as a weapon if needed”. (participant #8)

**Table 3.** Examples of Psychological Contexts

** Example about self confidence** Example 7: “When walking outside, the stick does not stand in the way. It gives her more confidence with walking”. (participant #22)
** Example about remaining independent and self-sufficient** Example 8: “The wheeled walker gives support and she can go on doing her shopping independently. As a result, she remains in a good mental state”. (participant #21) Example 9: “Not to be too much dependent on others, and as a result (she) can remain independent”. (participant #14)
** Example about feeling secure** Example 10: “(The wheeled walker) gives a sense of security and takes away the uncertainty when walking”. (participant #7)
** Example about fear of falling** Example 11: “In order to give this woman more support and to avoid any possible fear of falling again, I will advise her to use one or two crutches”. (participant #35)

## Footnotes

1 Available from http://www.qsrinternational.com.

2 Low = up to and including lower technical and vocational training; Medium: up to and including secondary technical and vocational training; High: up to and including higher technical and vocational training and university.

## References

[1] Kivits J (2004). Researching the 'Informed Patient' the case of online information seekers. Information, Communication & Society.

[2] Ferguson T (1997). Health online and the empowered medical consumer. Jt Comm J Qual Improv.

[3] Durnell Cramton C (2001). The Mutual Knowledge Problem and Its Consequences for Dispersed Collaboration. Organiz Sci.

[4] Te'eni D (2001). Review: A cognitive -affective model of organizational communication for designing IT. MIS Quarterly.

[5] Te'eni D, Schwartz DG (2000). Contextualization in Computer-Mediated Communication: Theory informs design. Inf Sys Rev.

[6] Alpay LL, Toussaint PJ, Ezendam NPM, Rövekamp T, Graafmans W, Westendorp R (2004). Easing internet access of health information for the elderly users: design considerations and usability. Health Informatics J.

[7] Alpay LL, Toussaint PJ, Ezendam NPM (2007). The Dutch website "SeniorGezond": An illustration of a road map for the informed patient. Managed Care. 2007. Available fromhttp://www.forummanage-dcare.ch (Publikationen 2007, Nr. 2) or http://www.tellmed.ch (Fachliteratur, Managed Care Nr.2 2007).

[8] Samelson EJ, Zhang Y, Kiel DP, Hannan MT, Felson DT (2002). Effect of birth cohort on risk of hip fracture: Age-specific incidence rates in the Framingham Study. Am J Public Health.

[9] Witkin HA, Ottman PK, Raskin E, Karp SA (1971). A manual for the embedded figures tests.

[10] Perkins D, Wiske MS (1998). What is understanding?. Teaching for Understanding.

[11] Bloom B (1956). Taxonomy of educational objectives: the classification of educational goals: Handbook 1, Cognitive Domain.

[12] Kim KS, Mariani J, Harman D (2000). Individual differences and information retrieval: implications on Web design.

[13] Eremenco S, Cella D (2005). A comprehensive method for the translation and cross-cultural validation of health status questionnaire. Eval Health Prof.

[14] Weinstein N, Lyon J, Sandman P, Cuite C (1998). Experimental evidence for stages of health behavior change: The precaution adoption process model applied to home radon testing. Health Psychol.

[15] Armitage C, Conner M (2000). Social cognition models and health behaviour: A structured review. Psychol Health.

[16] Ford N, Miller D, Moss N (2001). The role of individual differences on Internet searching: an empirical study. J Am Soc Inf Sci.

[17] Attfield S, Adams A, Blandford A (2006). Patient information needs: pre and post consultations. Health Informatics J.

[18] Wilson T (1981). On user studies and information needs. J Doc.

[19] Gilbert DT, Tesser A (1995). Attribution and interpersonal perception. Advanced social psychology.

[20]  Sherif C, Sherif M, Nebergall R (1965). Attitude and attitude change: The social judgment-involvement approach.

[21] Luken P (1987). Social Identity in Later Life: A Situational Approach to Understanding Old Age Stigma. Int J Aging Hum Dev.

[22] Kelly D, Cool C (2002). The effects of topic familiarity on information search behavior.

[23] Bandura A (1997). Self-efficacy: The exercise of control.

[24] Hjørland B (2000). Information seeking behaviour. What should a general theory look like?. New review of Information Behaviour research.

[25] Martzoukou K (2005). A review of web information seeking research: considerations of method and foci of interest. Information Research.

